# The Ionospheric view of the 2011 Tohoku-Oki earthquake seismic source: the first 60 seconds of the rupture

**DOI:** 10.1038/s41598-020-61749-x

**Published:** 2020-03-23

**Authors:** Mala S. Bagiya, Dhanya Thomas, Elvira Astafyeva, Quentin Bletery, Philippe Lognonné, Durbha Sai Ramesh

**Affiliations:** 1 Indian Institute of Geomagnetism (DST), Navi Mumbai, India; 2 Institut de Physique du Globe de Paris, Université de Paris, CNRS UMR 7154 Paris, Cedex France; 3 Université Côte d’Azur, IRD,CNRS, Observatoire de la Côte d’Azur, Géoazur, Sophia-Antipolis, Valbonne, France

**Keywords:** Natural hazards, Solid Earth sciences, Space physics

## Abstract

Using the specific satellite line of sight geometry and station location with respect to the source, Thomas *et al*. [Scientific Reports, 10.1038/s41598-018-30476-9] developed a method to infer the detection altitude of co-seismic ionospheric perturbations observed in Global Positioning System (GPS) – Total Electron Content (TEC) measurements during the Mw 7.4 March 9, 2011 Sanriku-Oki earthquake, a foreshock of the Mw 9.0, March 11, 2011 Tohoku-Oki earthquake. Therefore, in addition to the spatio-temporal evolution, the altitude information of the seismically induced ionospheric signatures can also be derived now using GPS-TEC technique. However, this method considered a point source, in terms of a small rupture area (~90 km) during the Tohoku foreshock, for the generation of seismo-acoustic waves in 3D space and time. In this article, we explore further efficacy of GPS-TEC technique during co-seismic ionospheric sounding for an extended seismic source varying simultaneously in space and time akin to the rupture of Mw 9.0 Tohoku-Oki mainshock and the limitations to be aware of in such context. With the successful execution of the method by Thomas *et al*. during the Tohoku-Oki mainshock, we not only estimate the detection altitude of GPS-TEC derived co-seismic ionospheric signatures but also delineate, for the first time, distinct ground seismic sources responsible for the generation of these perturbations, which evolved during the initial 60 seconds of the rupture. Simulated tsunami water excitation over the fault region, to envisage the evolution of crustal deformation in space and time along the rupture, formed the base for our model analysis. Further, the simulated water displacement assists our proposed novel approach to delineate the ground seismic sources entirely based on the ensuing ionospheric perturbations which were otherwise not well reproduced by the ground rupture process within this stipulated time. Despite providing the novel information on the segmentation of the Tohoku-Oki seismic source based on the co-seismic ionospheric response to the initial 60 seconds of the event, our model could not reproduce precise rupture kinematics over this period. This shortcoming is also credited to the specific GPS satellite-station viewing geometries.

## Introduction

The seismic rupture of the Mw 9.0 March 11, 2011 Tohoku-Oki earthquake (henceforth the Tohoku-Oki earthquake) was rather complex. Numerous efforts, using various approaches and different datasets of near and far field observations, have been devoted to studying the source characteristics of the Tohoku-Oki earthquake (see review article by Lay^1^ and references therein). The earthquake initiated at ~29 km depth (https://earthquake.usgs.gov), half-way between the trench and the coast, and moved towards the trench with further propagation in the north and south directions along the trench within ~150 s of the event^2^. The crustal deformation generated by subduction earthquakes is challenging to monitor as the deformation is mainly distributed offshore. The Tohoku-Oki earthquake is an exceptional case of study as it was recorded by an unprecedented amount of high-quality complementary data, including ocean-bottom geodetic instruments^3,4^ and deep-water tsunami sensor^5^. Joint inversion of these near-field observations, along with dense networks of far-field seismological and geodetic data, allowed to characterize the details of the co-seismic slip on the fault^2^.

The response of the overlying atmosphere to earthquakes significantly depends on the crustal deformation that evolves during the rupture process around the epicentral region^6–12^. Most of the existing literature assumes the maximum displacement along the rupture as a point source. While the point source approach is correct for small to moderate earthquakes, for great events this assumption might be erroneous/not correct. For instance, Heki *et al*.^6^ considered multiple ground sources along the ~1300 km long fault segment which ruptured during the Mw 9.1 December 26, 2004 Sumatra earthquake to explain the co-seismic ionospheric variations due to this event and demonstrated that the response reflected the contribution of each sub-source. Coisson *et al*.^13^ have also shown that the amplitude of the tsunami-driven ionospheric signal requests the use of a finite source model, as a point source is overestimating the amplitude. This analysis was later confirmed by Rakoto *et al*.^14,15^.

The ionospheric response to the Tohoku-Oki earthquake has been extensively studied on both regional (near field) and global (far field) scale^8,13–23^. Most of these studies were performed using Global Positioning System (GPS) - Total Electron Content (TEC) observations. Astafyeva *et al*.^8^ used 1 Hz GPS-TEC data to study the ionospheric perturbations over the seismic fault region. Such high-rate data allowed to determine the first arrivals of co-seismic ionospheric perturbations with high precision and to obtain, for the first time, ionospheric images of seismic fault. They were able to detect the perturbations as early as ~464 s after the event over the rupture area.

Interestingly, from 1 Hz GPS-TEC measurements, Thomas *et al*.^24^ presented the first detection of co-seismic ionospheric perturbations (CIP) as early as ~411 s after Mw 7.4 March 9, 2011 Sanriku-Oki earthquake. Further, based on the modest 3D acoustic ray tracing model they inferred, for the first time, the detection altitudes of these early measured CIP in GPS-TEC. They suggested that CIP detection right over the source using low satellite elevation geometry with the GPS station and source laying in the same azimuth plane provides an opportunity to detect the CIP at lower ionospheric altitudes. Earlier than this report, GPS-TEC technique was best known for deriving the spatio-temporal evolution of CIP near as well as far field of the epicentre. Based on the method by Thomas *et al*.^24^ it is now possible to estimate the detection altitude of GPS-TE1C measured CIP. However, this study was performed for a much smaller rupture area than the Tohoku-Oki mainshock with a maximum uplift of ~0.3 m and thus could be considered as a point source.

Here, we extend the method and analysis proposed by Thomas *et al*.^24^ to the Tohoku-Oki event with an aim to assess the further utility of GPS-TEC technique for co-seismic ionospheric sounding during such great event with the complex source varying simultaneously in space and time. For the first time, we estimate the detection altitudes of the early measured ionospheric signatures during this earthquake. Based on the azimuthal distribution of early detected CIP we delineate, for the first time, distinct seismic sources that evolved during the initial 60 s of the event and responsible for the generation of the observed CIP pattern over the Tohoku-Oki rupture area. Simulated tsunami water excitation over the fault region served as the base for this investigation. Moving a step further, we could trace the ground seismic sources entirely based on the ensuing ionospheric perturbations which were not well indicated by the simulated tsunami water excitation within this stipulated time. This novel finding could be considered as an important contribution to the challenging objective of identifying seismic source characteristics from the ionosphere.

The obtained segmentation of the Tohoku-Oki seismic rupture into multiple seismic sources during the initial 60 s of the event is discussed in view of the low elevation observing satellite geometry and widely spread GPS receivers over the Japan region. It should be noted that despite providing reasonably precise segmentation of an extended seismic source, our analysis failed at reproducing the rupture kinematics within this stipulated time. This aspect is also attributed to the specific observing station-satellite geometries.

## Early observed co-seismic ionospheric perturbations during the Tohoku-Oki earthquake

The evolution of CIP depends on the propagation of seismo-acoustic waves in the atmosphere and their further interactions with the ambient ionospheric plasma. Since electron density starts to increase at ionospheric altitude of ~100 km and upwards, the CIP can evolve near and beyond these altitudes. However, the total ionisation density is smaller at lower ionospheric altitudes (~100–150 km) and thus the amplitude of CIP may not be significant.

In general, ionospheric perturbations are considered to manifest best at the maximum electron density altitude^25^. TEC represents the integrated electron density along the satellite-receiver line of sight (LOS) and thus detection altitudes of GPS-TEC measured ionospheric perturbations are not known. In view of these, the GPS-TEC derived ionospheric perturbations are mostly considered at the maximum electron density altitude^26,27^.

**Figure 1:**
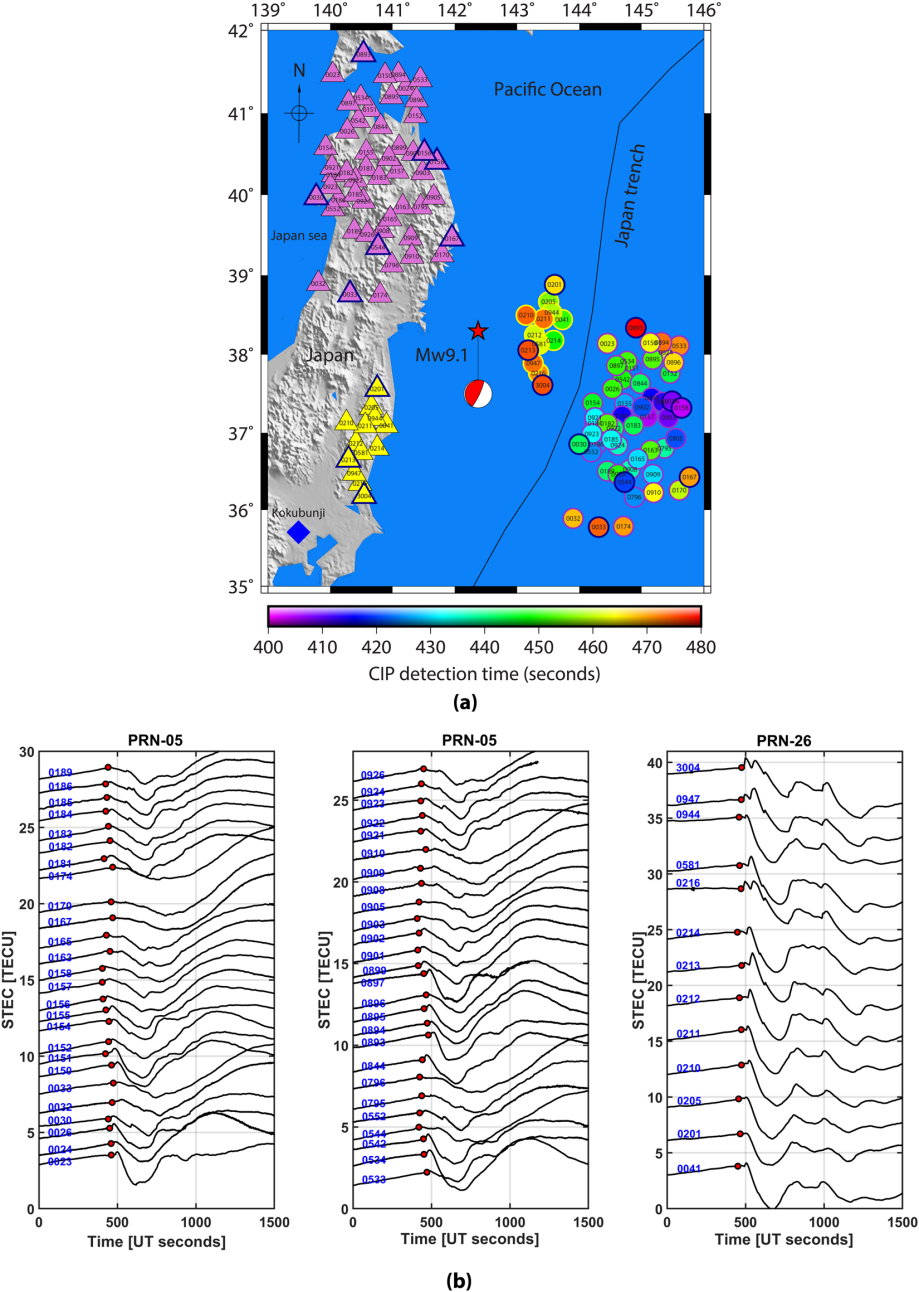
(**a**) Spatial evolution of CIP, as observed by PRN 05 and PRN 26, estimated at 250 km of ionospheric piercing point altitude within 480 seconds of the Tohoku-Oki event. The triangles show the observing station coordinates derived using Precise Point Positioning (PPP) mode. The stations that recorded the CIP observed by PRN 05 are highlighted in pink triangles and that recorded the CIP observed by PRN 26 are highlighted in yellow triangles. The CIP are also outlined accordingly. CIP highlighted in blue are further presented using our 3D model analysis. The figure is prepared using the GMT 5.4.1.^43^ (**b**) Temporal evolution of CIP shown in 1(a). The time axis is zeroed with respect to the earthquake onset time. Each CIP time series is labelled with a respective recording station id. The onset of CIP is denoted with the red disk.

Considering a CIP evolution near the peak density altitude, we present the spatio-temporal evolution of CIP, within 480 s of the event, at an altitude of 250 km over the Tohoku-Oki epicentral region in Fig. 1a. The peak density altitude information is derived based on the International Reference Ionosphere (IRI)–2016 model^28^ for the event date, time and epicentre location. It should be noted that only PRN 05 and 26 could observe the CIP within this stipulated time. The temporal evolution of these CIP is presented in Fig. 1b. CIP onset is marked with a red disk in each time series. These time series are enlarged around the CIP detection times, between 250 to 750 s, and included in the supplementary information as figure Suppli_1 for further reference. Figure 1a,b illustrate that the CIP started appearing at ~404 s of the event.

We model the space and time evolution of seismo-acoustic rays at 250 km of altitude considering the epicentre as a source (figure Suppli_2a). The rays are modelled from the earthquake onset time and they require around 600 s or more to arrive at an altitude of 250 km. Thus, if the CIP had to evolve near the ionospheric peak density altitude then they would have appeared at and after ~600 s in GPS-TEC during the Tohoku-Oki event. However, they started to appear ~404 s after the event and thus it is obvious that CIP shown in Fig. 1 are certainly not evolved at ionospheric peak electron density altitude. These CIP are described as early observed CIP. We restrict the CIP evolution to 480 s in Fig. 1. This aspect is discussed later in the text.

## Delineation of multiple seismic sources based on the azimuthal distribution of early observed CIP around the Tohoku-Oki epicentral region

Near field co-seismic ionospheric response to an earthquake largely depends on the crustal deformation around the epicentre. For small to moderate earthquakes, the crustal deformation area is more confined and the rupture duration is also shorter than for large ones. For instance, during the Mw 7.4 Sanriku-Oki earthquake, the rupture lasted for ~55 s and the extent of the rupture was ~90 km^29^. However, in the case of great earthquakes, a larger area may rupture over a longer duration. The Tohoku-Oki earthquake rupture lasted about ~150 s with most of the moment energy released between 40 to 90 s and the total fault extended over the area of ~400 × 200 km^1,2,30^. Therefore, the crustal deformation during the Tohoku-Oki event cannot be assumed as a point source, as was in the case of the Mw 7.4 Sanriku-Oki earthquake^24^. The information on the spatio-temporal evolution of the crustal deformation during such events becomes vital while studying the subsequent ionospheric perturbations over the source region.

Since the Tohoku-Oki earthquake was an offshore event, we simulate the ocean surface displacement surrounding the Tohoku-Oki epicentre and use it as a proxy to envisage the crustal deformation. The ocean surface displacement, CIP onset detection and our model analysis are considered from the earthquake onset time (https://earthquake.usgs.gov) for better comparison.

**Figure 2:**
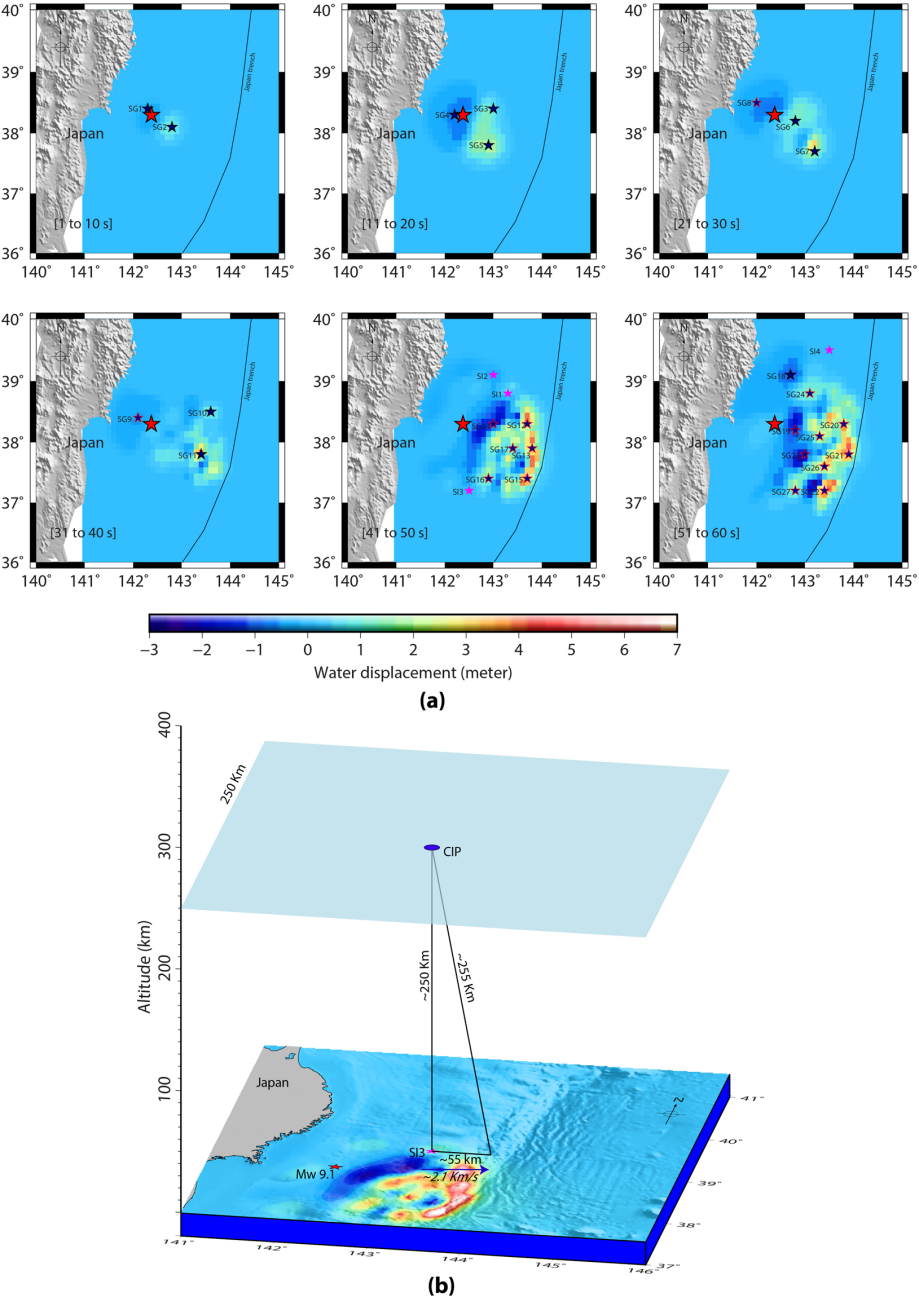
(**a**) Simulated tsunami water excitation within 60 s of the Tohoku-Oki event summed at every 10 s. The red star denotes the earthquake epicentre. Blue stars denote the maximum water displacement fields (i.e. uplift or subsidence) separated by ≥0.5° in each 10 s window. The blue stars highlighted in black are the sources that could generate CIP within 480 s of the event onset. The sources highlighted in red could not produce any CIP within this stipulated time. Magenta stars are the sources that identified entirely from the ionosphere and simulation could not reproduce any significant ocean water displacement surrounding these locations. (**b**) Geometrical model demonstrating the uncertainty in CIP onset time due to a source movement within ~0.5^o^/~55 km of distance. A realistic scenario for the CIP generation at an ionospheric altitude of ~250 km by the overhead propagating seismo-acoustic ray from SI3 source is shown. If the source propagates by ~55 km at the ground with an average rupture velocity of ~2.1 km/s then the seismo-acoustic ray has to travel ~255 km of distance to generate this CIP and thus the ray propagation time increases accordingly. Please refer text for more details on this. The figure is prepared using the GMT 5.4.1^42^.

We compute the ocean surface displacement every 10 s from the earthquake onset to 60 s predicted by the source model of Bletery *et al*.^2^ (Fig. 2a). The displacement fields, i.e. uplift or subsidence, within the simulated water deformation patches separated by ≥0.5° either in latitude or longitude in each 10 s window are considered as likely seismo-acoustic sources. These are indicated as blue stars in Fig. 2a. From our model analysis, the sources highlighted with black outline could produce the CIP within 480 s of the event detected by PRNs 05 and 26. While no CIP were found to be generated by the seismic sources highlighted in red outline within this stipulated time. This aspect is discussed in terms of the limitations of this analysis later in the text.

In Fig. 2a, each source is labelled in chronological order with prefix ‘SG’ for easier visualisation. Here, ‘SG’ describes the seismo-acoustic sources demarcated based on the ocean surface displacement. The sources highlighted with magenta stars are the major outcome of this study as they are identified entirely from the ionosphere. These sources are labelled in chronological order with ‘SI’ prefix. It should be noted that, unlike the 'SG' sources, the 'SI' sources do not show any significant sea water displacement. However, all SI sources are located in certain water displacement area, except the SI4 where the simulation could not reproduce any ocean surface excitation. Nevertheless, the evolved ionospheric perturbations entailed the presence of SI4 seismic source.

We model the simultaneous propagation of seismo-acoustic rays from the demarcated SG at the initial time of each 10 s window. That is, the seismo-acoustic ray propagation from SG1 and SG2 is considered at the event onset time, from SG3, SG4 and SG5 at 11 s of the event, likewise further. Therefore, the ray propagation from the sources demarcated in a given time window may contain the temporal uncertainty of 10 s.

To identify the contribution of each of these sources in generating CIP (Fig. 1), we use the method developed by Thomas *et al*.^24^ that mainly estimates the actual detection altitudes of CIP measured by GPS. It should be recalled that in the case of GPS measured CIP, the detection time of CIP is recorded but the detection altitude is not known. Therefore, time-dependent inversion of CIP similar to the analysis of Bletery *et al*.^2^ cannot be performed to identify the ground seismic sources of distinct CIP. Based on the (i) satellite LOS and station geometry with respect to the source and (ii) spatio-temporal evolution of CIP surrounding the epicentre, we not only estimate the detection altitude of CIP but extend the efficacy of the method by Thomas *et al*.^24^ to classify the distinct seismic sources that evolved during the initial 60 s of the rupture and generated the CIP (Fig. 1). That is the atmospheric response to seismic energy released during the first 60 s of the Tohoku-Oki earthquake and our primary interest here.

The first CIP after the Tohoku-Oki event was detected within ~404 s (Fig. 1). Therefore, the CIP detected after 60 s of this possibly generated by the seismic sources evolved in 60 s of the event. However, this consideration is valid in light of the followings. The overlying atmosphere, which hosts the seismic energy, should be uniformly stratified within 60 s of the event. Since the demarcated sources are spread over ∼3.2° of latitudes (36.8° to 40° N) and ∼3° of longitudes (141° to 144°E), it is assumed that the atmosphere may not vary significantly over this smaller region. Further, the satellite LOS should propagate overhead the source at the respective CIP detection time. In other words, the azimuthal separation between the station-satellite LOS plane and the station-source plane should be minimal.

Though the demarcated sources are preferred at the area of maximum or minimum water displacement within a patch, the displacement that evolved anywhere within a given patch can act as a source of CIP. Therefore, the CIP evolution time highly depends on the source kinematics within the patch and thus we account for the respective source mobility along the rupture in a given patch. We propose a modest geometrical model to estimate the corresponding time uncertainty (Fig. 2b). In Fig. 2b, the seismo-acoustic rays, from a given source, require ~513 s to propagate vertically upward at the peak electron density altitude of ~250 km. This propagation time is estimated using an average acoustic velocity of ~487 m/s (figure Suppli_2c). Considering a deformation patch of ~0.5°, the source of CIP can be anywhere within the lateral distance of ~55 km in a given patch. Thus, if a source moves by 55 km then the seismo-acoustic rays need to travel the vertical distance of ~255 km to arrive at the detection location of this specific CIP and the estimated wave propagation time is ~523 s (Fig. 2b). The Tohoku-Oki rupture propagated with minimum to maximum velocity of 1.1 km/s to 3.1 km/s^2^. We consider the average rupture propagation velocity of ~2.1 km/s for a lateral source movement of ~55 km which accounts for the time period of ~26 s. Therefore, for this moving source, the maximum time uncertainty in the CIP evolution can be around ~16 s. That is, ~513 s (time requires for seismo-acoustic rays to travel at ~250 km atmospheric altitude if source is located at the edge of the water deformation patch) + 26 s (time requires for a source to move ~55 km at the ground within a given water deformation patch and to reach another edge) – ~523 s (time requires for seismo-acoustic rays to travel at ~255 km atmospheric altitude when source moves by ~55 km i.e. another edge). Based on this, we update the CIP detection time of ~464 s by the uncertainty of ~16 s and present the CIP evolution from the first CIP onset (i.e. ~404 s of the event) to 480 s of the event in Fig. 1.

We now attempt to delineate the contribution of individual seismic sources (Fig. 2a) in generating the CIP based on the estimation of their actual detection altitudes. We execute this analysis for each CIP shown in Fig. 1. However, the model output is presented for the CIP highlighted with a blue outline. The outcome for the remaining CIP are listed in supplementary Table 1 and mentioned in the text suitably.

**Figure 3:**
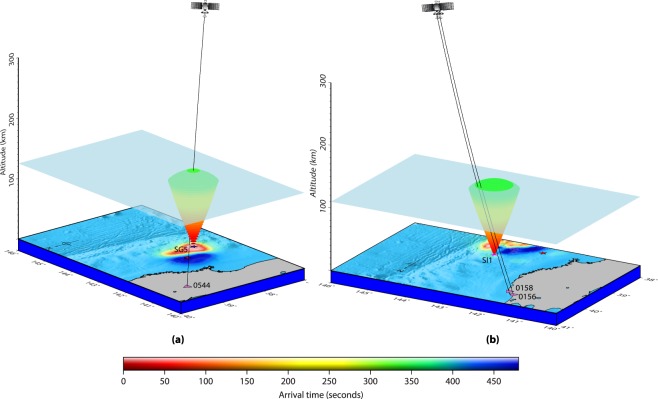
3D model identifying the seismic sources of CIP detected by PRN 05 from GPS stations of (**a**) 0544 and (**b**) 0156 and 0158. The evolution of seismo-acoustic rays is shown in 3D space and time. Triangles represent the GPS stations. The satellite is illustrated in the orbital plane of ~22,000 km (not to the scale). The first interaction of PRN 05 LOS from 0544 station and seismo-acoustic rays from SG5 source could be noticed in (**a**). The interaction occurs at ~126 km of altitude within ~420 s of the event. The interaction altitude is represented with a transparent 3D plane for easier visualization. PRN 05 LOS from 0156 and 0158 GPS stations interacted first with the seismo-acoustic rays propagating from SI1 source. For PRN 05–0158 station combination the interaction between the satellite LOS and seismo-acoustic rays occurs at ~112 km altitude within ~433 s of the event while for that of the PRN 05–0156 station combination it occurs at ~114 km altitude within ~437 s of the event. The figure is prepared using the GMT 5.4.1^43^.

Firstly, we model the seismo-acoustic rays propagation in 3D space (latitude, longitude and altitude) and time from the SG sources (Fig. 2a). The ray propagtion is modelled from the respective source onset times (as described earlier). The spatio-temporal evolution of seismo-acoustic rays from SG sources are then examined at every instant of time for their possible interaction with the LOSs of PRN 05 and PRN 26 from GPS stations shown in Fig. 1. It could be noticed that PRN 05 LOS from 0544 GPS station interacted first with the seismo-acoustic rays propagating in the atmosphere after the Tohoku-Oki event. While tracing back to the generative seismic source, it has been found that these rays were produced by the SG5 (Fig. 2a). Our model demonstrating the evolution of seismo-acoustic rays from SG5 source that evolved at 11 s of the rupture is presented in Fig. 3a. The figure also demonstrates the first interaction between the PRN 05 LOS from GPS station 0544 and the propagating seismo-acoustic rays from SG5. The interaction altitude of ~126 km is highlighted with a transparent 3D plane for easier visualisation. The LOS is estimated based on the satellite navigation data and receiver coordinate. Interestingly, the modelled interaction time of ~420 s significantly corroborates the CIP detection time by PRN 05 from this station (Fig. 1). The azimuthal difference between the station (0544) – source (SG5) plane and the station – satellite LOS plane was ~0.05° at this time. Thus, our model analysis conferred that PRN 05 LOS from 0544 station first interacted with the seismo-acoustic rays propagating overhead SG5.

From figure Suppli_3b, the seismo-acoustic rays propagating overhead require ~409 s to arrive at the ionospheric altitude of ~126 km. However, our model demonstrates this altitude detection at ~420 s. It has to be recalled that CIP detection times in Fig. 1 and modelling of the rays from multiple seismic sources are conferred with respect to the event onset time. Subsequently, the rays in Fig. 3a are computed after 11 s of the event. Since the CIP as observed by PRN 05 from 0544 station is plausibly generated by SG5, the actual onset time of this CIP could be ~420–11 ~ 409 s which corroborates well with the modelled arrival time of vertically propagating seismo-acoustic rays at altitude of ~126 km (Fig. 3a and Suppli_3b).

While, PRN 05 LOS from 0544 GPS station interacted first with the seismo-acoustic rays propagating after the event, the first CIP after the event was detected by PRN 05 from 0158 GPS station. This CIP was detected within ~404 s of the event. Interestingly, PRN 05 LOS from 0158 station could not interact with the seismo-acoustic rays propagating from any of the SG sources despite introducing the time difference of 60 s with respect to the actual CIP detection time. The time difference of 60 s is understood in the following way.

The interaction between the satellite LOS and propagating seismo-acoustic rays is considered under two conditions. First of all, the interaction should occur ~110 km and above of atmospheric altitudes. Thus, sufficient electron density for the evolution of ionospheric perturbations can be found. The next criterion is, station-satellite LOS plane should not be very far from the station-source plane. The satellite LOS plane with growing azimuthal difference from a station-source plane would interact with the propagating seismo-acoustic rays at higher inclinations i.e. away from the source zenith where atmospheric horizontal wind effects may become more significant^24,31^. Thomas *et al*.^24^ estimated the maximum difference up to ~51 s between the modelled arrival time of the rays and observed detection time of CIP in GPS-TEC. This time difference was derived for the azimuthal difference up to ≤15° between the station-satellite LOS plane and station-source plane. They attributed this difference to the NRLMSISE-00 model^32^ derived atmospheric temperature and density parameters that were used to compute the acoustic wave velocity profile and the horizontal winds. They observed that increasing azimuthal difference between the station-satellite LOS and station-source planes accelerates the difference between the modelled ray arrival time and observed detection time which does not allow to estimate the precise detection altitudes. Since our aim is to identify the generative source of each CIP, as precisely as possible, we prefer that the satellite LOS should not be too far from the station-source plane. Therefore, most of the satellite LOSs presented in this study maintain the azimuthal difference of ≤15° with respect to the station-source plane, the difference for which Thomas *et al*.^24^ estimated the temporal difference of ~51 s. However, this temporal difference also depends on the respective station-source distance. We adopt this estimated time difference and the temporal uncertainty of 10 s during the simultaneous propagation of seismo-acoustic rays from the demarcated sources in each time window (as described earlier) and implement the maximum temporal difference up to ~60 s while modelling the rays.

**Figure 4:**
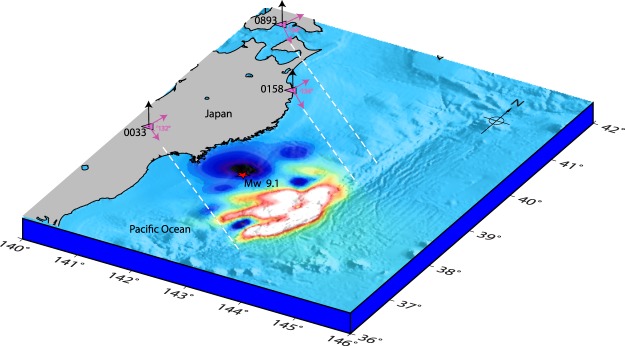
Realistic schematic showing PRN 05 azimuthal geometry from three GPS stations at their respective CIP recording times. Pink arrows denote the corresponding azimuth angle. GPS stations 0893 and 0158 were located north-north-west of the epicentre while 0033 was located in the west-west-north. The dashed white lines show the projection of PRN 05 LOS over the fault region, from the respective station. The figure is prepared using the GMT 5.4.1^43^.

Despite modelling the rays up to ~464 s (404 s + 60 s) of the event, we could not identify suitable seismic source of the CIP detected by PRN 05 from 0158 station. From Fig. 1, 0158 GPS station was located north-north-west of the epicentre and the azimuth of PRN 05 LOS from this station at CIP detection time was ~134°. The realistic 2D schematic of Fig. 4 contents with the epicentre location, 0158 GPS station geometry with respect to the epicentre and PRN 05 LOS azimuthal geometry with respect to 0158 station could be seen for more clarity on this. The dashed white line shows the projection of PRN 05 LOS over the fault region. From Fig. 4, PRN 05 LOS could possibly interact with the rays propagating from a seismic source located somewhere north or east of the epicentre. However, the SG demarcated to the north of the epicentre (Fig. 2a) could not explain this. Thus, we search for the additional seismic sources in the north. From Fig. 2a, northward evolution of SG was significant after 40 s of the event. Accordingly, we extend our analysis to the ocean surface displacement appeared north of the demarcated SG sources and east-north-east and north-north-east of the epicentre respectively in the 41–50 s and 51–60 s time windows. We presume that these displacement fields are the probable sources of seismo-acoustic rays and prefer that they remain separated by ≥0.5° from the demarcated SG. Based on this, we could demarcate two seismic sources north-north-east and north-east of the epicentre in the 41–50 s time window. It should be noted that these sources have been identified entirely based on the station-satellite LOS geometry, with respect to the epicentral region, that detects the ionospheric perturbations. In this context, these sources are labelled as SI1 and SI2 in Fig. 2a.

We model the evolution of seismo-acoustic rays from SI1 and SI2 sources and examine their interaction with the PRN 05 LOS from 0158 GPS station at every instant of time. Our model analysis suggests that the LOS interacted with rays propagating from SI1 source within ~433 s of the event at ~112 km of altitude (Fig. 3b). However, this LOS observed the CIP within ~404 s of the event. Further, within 4 s of this interaction, PRN 05 LOS from 0156 GPS station interacted with the seismo-acoustic rays and source was indeed the SI1. PRN 05 LOS from 0156 station and its first communication with rays evolving from source SI1 are also demonstrated in Fig. 3b. In the case of 0156 GPS station, the interaction occurred at ~437 s of the event which projects the difference of ~31 s with that of the observed CIP detection time (~408 s) by PRN 05 from this station. The modelled detection altitude for this CIP is ~114 km. Due to a very small difference between the modelled detection altitudes of these two CIP, it is not possible to demarcate them separately in the figure. Thus, we highlight the altitude of ~112 km (detection altitude in the case of 0158 GPS station) with a transparent 3D plane. The GPS stations 0158 and 0156 are located at a respective distance of ~224 km and ~245 km from source SI1. Both the LOS captured the CIP at an elevation angle of ~25°.

Further, the azimuthal separation between the station – source (SI1) plane and satellite LOS plane for station 0158 is ~8° while for 0156 is ~7° which convey that both the LOSs interacted with the rays a little away from the zenith of the source. This could be easily visualised from Fig. 3b. The residual difference of ~31 s between the modelled ray arrival times and observed CIP detection times are thus attributed to the respective azimuthal separation as discussed earlier in view of the arguments presented by Thomas *et al*.^24^. Interestingly, the azimuthal separation is relatively less in the case of PRN 05–0156 station but still the CIP measurement time and estimated detection altitude are higher compared to that of the PRN 05–0158 station. This is due to the difference in the respective station distance from source SI1. GPS station 0156 is located more distant from the SI1 compared to that of the 0158 station. This aspect is discussed separately later.

Concerning the evolution of CIP at ionosphere altitude of ~112 km and nearby, we would like to mention that strong sporadic-E layer was recorded by Kokubunji ionosonde^33^ during the earthquake occurrence time. The location of the ionosonde is shown in Fig. 1 with blue diamond. The ionograms from Kokubunji station during earthquake period, 15 min before the event and 15 min after are shown in figure Suppli_3. The presence of the sporadic-E layer during the earthquake occurrence period favours the evolution of CIP at lower ionospheric altitudes. It should be noted that the altitudes in ionograms are virtual heights and the real heights are lesser than these. The relatively tiny CIP amplitudes at their onset times (Fig. 1b) demonstrate that the CIP were evolved in lesser electron density region.

**Figure 5:**
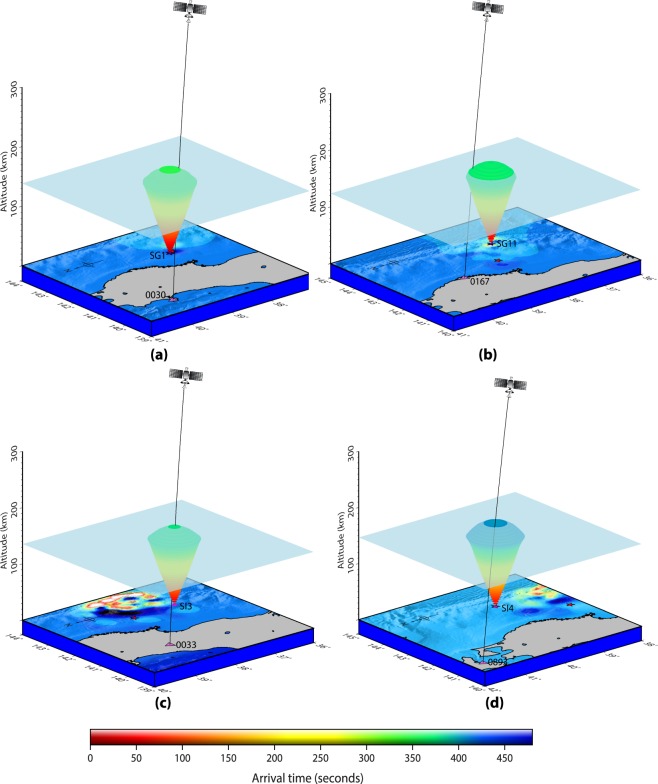
3D model identifying the seismic sources of CIP observed by PRN 05 from (**a**) 0030, (**b**) 0033, (**c**) 0167, and (**d**) 0893 GPS stations. From our model analysis, these CIP are induced by SG1, SI3, SG11, and SI4 respectively. The first interactions between the PRN 05 LOS from these stations and the rays from SG1, SI3, SG11 and SI4 respectively at ~441 s, ~473 s, ~469 s and ~509 s at the respective altitude of ~138 km, ~135 km, ~121 km and ~141 km are shown. Other information is the same as Fig. 3. The figure is prepared using the GMT 5.4.1^43^.

We extend our model analysis for the remaining PRN 05 measured CIP of Fig. 1 and present the results for the GPS stations of 0030, 0167, 0033 and 0893 in Fig. 5. From Fig. 1, these stations are distributed over a wide azimuthal region and thus the CIP recorded from these stations may be suitable to identify the seismic sources spread accordingly. From Fig. 5a, the first interaction between PRN 05 LOS from 0030 GPS station and seismo-acoustic rays occurred at ~442 s of the event. The ray source is delineated as SG1 (Fig. 2a). The estimated modelled arrival time of ~442 s, at the interaction epoch, concurs well with the actual CIP detection time by PRN 05 from this station. The modelled interaction altitude is ~139 km. In the case of 0167 GPS station, the first interaction between PRN 05 LOS and seismo-acoustic rays occurred at ~470 s and our model suggests that these rays are evolved from SG11 (Fig. 2a). The modelled interaction altitude is ~123 km. Despite the azimuthal separation of ~11° between the station (0167) – source (SG11) plane and station – satellite plane, the modelled interaction time and observed CIP detection time corroborated well. This could be attributed to the relatively smaller station-source distance.

PRN 05 LOS from 0033 and 0893 GPS stations could not interact with the rays evolving from SG sources (Fig. 2a), even with the time difference of ~60 s with respect to each CIP detection times. So, we follow a similar strategy which we have proposed in the case of 0158 and 0156 GPS stations and identify the plausible seismic sources, based on the respective station and satellite LOS geometry with respect to the epicentral region. The azimuth of PRN 05 from 0033 station at the CIP detection time was ~132° (Fig. 4). The station 0033 was located west-north-west of the epicentre. Accordingly, the seismic source located south of the epicentre could be a potential candidate for generating the CIP measured by PRN 05. The realistic 2D schematic of Fig. 4 containing the 0033 GPS station geometry with respect to the epicentre and PRN 05 LOS azimuthal geometry with respect to 0033 station can be seen for this. The required seismic source can be explored as the ocean surface displacement evolved during 41–50 s farthest south of the epicentre. There was no water excitation before 41 s over this region (Fig. 2) and after 50 s, the temporal error exceeds our estimated error budget of ~60 s thus we have considered the displacement fields evolved during 41–50 s. We attempt to locate the sources based on our earlier consideration i.e. spatial separation between the sources should be ≥0.5° in a given time window of 10 s.

We then examine the interaction of PRN 05 LOS from 0033 GPS station and propagating seismo-acoustic rays at every instant of time. During these iterations, we have noticed that first interaction occurred at ~474 s of the event and the ray source is traced as the water displacement demarcated as SI3 in Fig. 2a. The modelled interaction time corroborated well with CIP detection time of ~474 s. The interaction altitude is estimated at ~136 km. Significant corroboration between the model estimated time and observed detection time could be understood in terms of a very small azimuthal separation (~0.2°) between the station (0033) – source (SI3) plane and the station-satellite LOS plane.

As similar to 0033, 0156 and 0158 GPS stations, difficulties were also observed in locating the generative seismic source of CIP detected by PRN 05 from 0893 station. Nevertheless, based on the 0893 station geometry with respect to the epicentre i.e. north-north-west and PRN 05 azimuthal geometry with respect to 0893 GPS station at the CIP detection time, the probable seismic source can be explored north-east of the epicentre. This could be verified from the 2D realistic schematic of Fig. 4. During the iterative run of our 3D acoustic ray tracing model, we could notice that PRN 05 LOS from 0893 station interacted with the seismo-acoustic rays at ~508 s of the event and the ray source is identified as SI4 which evolved during the 51–60 s of the event (Fig. 2a). Interestingly, the simulated ocean surface elevation did not show any water displacement at this location. However, our analysis argues the presence of a seismic source in terms of crustal deformation surrounding the SI4. Moreover, the CIP detection by PRN 05 from stations located in the farthest north i.e. 0894, 0533, 0895, 0024, 0896 and 0152 (Fig. 1) are best explained based on the seismic source of SI4. The detailed and complete model analysis for these CIP along with the remaining ones observed by PRN 05 and their respective delineated generative seismic sources are given in supplementary Table 1. The difference between the observed CIP detection times and modelled arrival times was found to be more for the larger azimuthal separation between station-satellite LOS plane and station-source plane and/or the station-source distance. The table also contains the estimated CIP detection altitudes.

**Figure 6:**
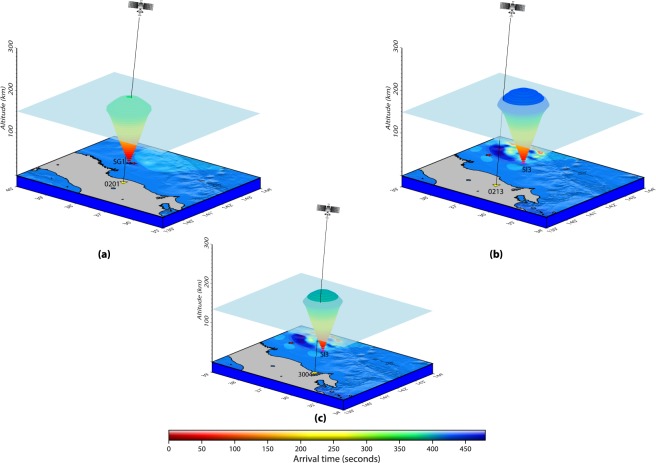
Identification of seismic sources for CIP observed by PRN 26 from (**a**) 0213, (**b**) 3004, and (**c**) 0201 GPS stations. The first interaction of PRN 26 LOS from 0213 and 3004 GPS stations occurred with the seismo-acoustic rays evolving from SI3 source while that from 0201 station occurred with the rays from SG3 source. The interaction time and altitude are respectively ~532 s and ~149 km in case of 0213 station, ~498 s and ~135 km in case of 3004 station, and ~466 s and ~152 km for 0201 station. Other information is the same as Fig. 3. The figure is prepared using the GMT 5.4.1^43^.

In Fig. 1, PRN 26 could also capture a few CIP within 480 s of the event from the stations located southwest of the epicentre. We attempt to explore the generative sources of these CIP as well. We run our 3D acoustic ray tracing model for the demarcated SG sources (Fig. 2) and scrutinize the first interaction of PRN 26 LOS from each one of these stations to seismo-acoustic rays evolving from the SG. The outcomes of our model analysis for PRN 26 are presented in Fig. 6 for stations 0201, 0213 and 3004 and also listed along with the remaining CIP in supplementary Table 1. PRN 26 captured the CIP at relatively higher elevation angles, i.e. ~39°, compared to that of PRN 05. In Fig. 6, PRN 26 LOS from 0201 GPS station interacted first with the seismo-acoustic rays evolving from source SG1 within ~466 s. This interaction time matches well with the observed CIP detection time by PRN 26 from this station. The interaction altitude is estimated at ~152 km. The azimuthal separation between the 0201 – SG1 plane and the PRN 26 LOS from 0201 station is ~2°.

Incidentally, PRN 26 LOS from GPS stations of 0213 and 3004 could not interact with the seismo-acoustic rays evolving from SG sources even after introducing the uncertainty of ~60 s in the modelled time. Similar was found to be correct in the cases of 0214, 0216, 0581, and 0947 GPS stations. Therefore, we run our ray tracing model for SI sources (Fig. 2a). Based on this, SI3 best explains the CIP detection by PRN 26 from these stations. However, the differences between the observed CIP detection times and the modelled interaction times were estimated to be larger in these cases and also maximum in the case of 0213 GPS station. We attribute this to the higher azimuthal separation between PRN 26 LOS plane and respective station-source plane in addition to the respective station distance from SI3. The outcome of model analysis provided in supplementary Table 1 can be referred.

So, both SG and SI sources are classified in light of their ionospheric imprints. During classifying the SG, we first model the seismo-acoustic rays from the assumed sources (i.e. water displacement fields) then examine their interactions with the realistic GPS LOS and based on this delineate the suitable seismic source of corresponding CIP. While in the case of SI, we consider first the GPS station and satellite LOS geometry, with respect to the epicentral region, that records the CIP and accordingly probe the source of seismo-acoustic rays along the fault region.

Our calculation of sea surface elevation is based on the co-seismic slip model inverted from the most complete dataset of broadband seismometers, accelerometers, static and high-rate GPS, sea-floor geodesy and tsunami observations in the literature^2^. This model may be seen as a refined solution of earlier (and later) slip models which considered different subsets of the data inverted in this study^1^. However, we find that the demarcated SI sources do not exhibit significant water surface displacement similar to SG, SI3 and SI4 in particular. This misfit may partly be due to inaccuracies in the ground source model we used. Even though this model is the most-constrained in the literature, it can be seen as a (physics-based) extrapolation of discrete surface measurements which are relatively distant from SG sources.

**Figure 7:**
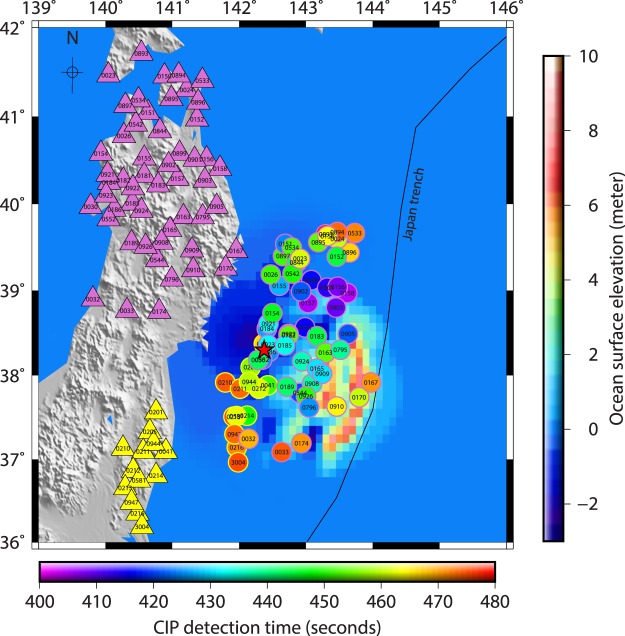
2D demonstration of the spatio-temporal evolution of CIP projected at their respective detection altitudes estimated in the present study (Supplementary Table 1). The total tsunami water excitation within 60 s of the Tohoku-Oki event is shown in the background for reference. Other information is the same as Fig. 1. Significant corroboration between the spatial evolution of water surface displacement and that of the CIP at their respective detection altitudes could be appreciated. The figure is prepared using the GMT 5.4.1^43^.

At the final step, we project the CIP distribution of Fig. 1a to their actual detection altitudes, estimated based on our 3D model analysis, and present the revised spatial evolution along with the total ocean surface displacement fields up to 60 s in Fig. 7. The reasonably good agreement between the CIP distribution and the total ocean surface displacement could be appreciated in sounding the rupture extent from the ionosphere during the initial 60 s of the Tohoku-Oki event. The ionospheric image after the Tohoku-Oki event reported by Astafyeva *et al*.^8^ was derived at fixed ionospheric piercing point altitude of 250 km. While the present study moves a step further and first estimates the respective CIP detection altitude in GPS-TEC and then derives the rupture extent from the ionosphere. Thus, provides a more accurate rupture extent.

Further, the present study reveals that the spatial evolution of CIP as detected by PRN 05 and PRN 26 within 480 s of the Tohoku-Oki event was not generated by a single seismic source but evidently by distinct seismic sources evolved along the rupture within 60 s of the event. Thus, our analysis based on the 3D acoustic ray tracing model and realistic GPS station-satellite LOS geometry could be considered as a fair alternative to segment an extended seismic source into multiple sources that can perturb the overlying atmospheric-ionospheric regimes during great earthquakes.

## Limitations of the proposed analysis

No CIP were linked to the SG developed further south and east of the epicentre near the Japan Trench within 480 s of the event. There could be two prime reasons for this: (i) the sources further south and east near the Japan Trench developed after 40 s of the event. From our analysis, PRN 05 and PRN 26 LOSs interacted first with the seismo-acoustic rays from sources developed earlier in time and closer to the epicentre (e.g. SG6, SG7, and SG11). However, the interaction of these LOSs with the rays propagating from the sources developed after 40 s of the event and away from the epicentre could not be denied after ~480 s of the event. Precisely due to this, we analyse the effects of rupture propagation up to 60 s of the event. However, future improvement of our model analysis for sources spaced more closely (<0.5°) may help in overcoming this shortfall. Anyways, suitable satellite LOSs should be available at an adequate time to detect the resultant CIP. (ii) If PRN 05 has to detect the seismic energy from the sources developed near the Japan Trench (e.g. SG21, SG22) within the above stipulated time, then GPS stations should be available further east of the Japan coast (e.g. east of station 0170, Fig. 1a), which is not the case here.

While deriving the rupture kinematics using the corresponding ionospheric perturbations, it has been noticed that due to the peculiar station-satellite LOS geometry with respect to the source, the addressed CIP evolution could not reproduce plausible kinematics of the Tohoku-Oki rupture in the stipulated time of 60 s. The supplementary movie 1 could be seen for more information on this. The first CIP was detected at ~404 s of the event from 0158 GPS station but this CIP was not generated by seismic source evolved at the earthquake onset time. However, the responsible seismic source was found to be located northeast of the epicentre and evolved after 40 s of the event. Due to the specific station geometry with respect to the source region, PRN 05 from 0158 GPS station could detect the first CIP after the event. Further, the seismic source SG1 was evolved at the earthquake onset time and the corresponding first CIP was observed by PRN 05 from 0186 GPS station within ~424 s of the event. From supplementary Table 1, PRN 05 from 0186 station detected the CIP almost overhead the SG1. However, this station was located relatively far from the source. In the case of increasing station-source distance, the corresponding altitude for interaction between the satellite LOS and the vertically propagating seismo-acoustic rays also increases. This fact could be verified from figure Suppli_2b. Two hypothetical stations along with the realistic satellite elevation geometry are presented at two different distances from a hypothetical source. The station located nearer to the source can observe the seismo-acoustic rays at relatively lower altitudes and thus early in time. Since PRN 05 from 0186 GPS station detected the CIP at a relatively higher altitude, the detection time was more compared to the CIP detection by PRN 05 from 0158 GPS station. It is pertinent to recall that we consider the interactions which occur ~110 km and above of atmospheric altitudes. Thus, stations located very near to the source might not be suitable to detect the CIP in stipulated time. In a nutshell, in addition to the GPS satellite geometry, station geometry is equally important while detecting the CIP. Thereby, the kinematics of the Tohoku-Oki rupture that evolved during the initial 60 s could not be well reproduced based on the corresponding ionospheric perturbations. Thus, there is a growing requirement of developing a method that can provide rupture kinematics on the basis of co-seismic ionospheric response.

## Conclusion

The Mw 9.0 March 11, 2011 Tohoku-Oki earthquake is studied with an aim to unfold the utility of GPS-TEC based co-seismic ionospheric sounding in revealing more on the seismic source characteristics. As this great offshore event occurred with an extended seismic source, varying both in space and time, the first challenge was to look for an alternative for crustal deformation variations during the rupture. The next face-off was to extend the efficacy of the method developed by Thomas *et al*.^24^ for an extended seismic source. Evidently, the present novel study could endure most of these challenges and tenders the methods (i) to identify the distinct seismic sources along the rupture and thus the segmentation of an extended seismic source (ii) to derive reasonably precise reflection of seismic rupture extent in the ionosphere in stipulated time besides cautioning about reproducing the kinematics of seismic rupture, based on the ensuing GPS-TEC measured ionospheric signatures (iii) to represent the spatio-temporal evolution of crustal deformation in terms of the ocean surface displacement fields surrounding the offshore epicentre.

## Methodology

### Estimation of the Ocean surface water column displacement during the Tohoku-Oki earthquake

We calculate the ocean surface elevation generated by the Mw 9.0, March 11, 2011 Tohoku-Oki earthquake. We use a kinematic source model of the co-seismic slip along the Japanese subduction fault inferred from geodetic, seismic and tsunami data^2^ to compute both the horizontal and vertical displacement field at the bottom of the ocean at every 1 s of the rupture. To do so, we consider the dislocation formulation of Savage (1980)^34^ in an elastic uniform half space, with elastic parameters depending on the sub-fault depth [Bletery *et al*.^2^, Figure S2, based on the 3D tomography of J-SHIS (http://www.j-shis.bosai.go.jp/map/) and Takahashi *et al*.^35^]. The depth of each sub-fault follows the slab1.0 model^36^ but is re-evaluated taking into account the bathymetry so that the fault reaches the surface at the trench^2,37^. We then calculate the resulting ocean surface elevation ${u}_{z}^{surface}(t)$ every 1 s of the rupture by summing the contributions of the vertical displacement of the ocean bottom ${u}_{z}^{bottom}(t)$ and the horizontal displacement (${{\rm{u}}}_{{\rm{x}}}^{{\rm{bottom}}}(t)$, ${u}_{y}^{bottom}(t)$) which, combined with steep bathymetry (such as in the Tohoku region), can produce strong additional water uplift^37^:

1 $${u}_{z}^{surface}(t)={u}_{z}^{bottom}(t)-\,\frac{\partial \beta }{\partial x}{u}_{x}^{bottom}(t)-\frac{\partial \beta }{\partial y}{u}_{y}^{bottom}(t)$$

where *β* is the bathymetry^38^. We then apply a $\frac{1}{\cosh (kh)}$ filter (where *k* is the wavenumber and *h* the water depth) to model the attenuation of the water column^39^ and sum the relative contributions of ${u}_{z}^{surface}(t)\,$from *t = 0* (origin time of the rupture) to t = 10 s, t = 20 s, t = 30 s, *…*, t = 60 s to obtain the ocean surface elevations at these times of rupture (Fig. 2a). These different steps are consistent with the calculation of the tsunami Green’s functions in Bletery *et al*.^2^, so that the sea surface deformation is consistent with the tsunami observations inverted in this study. Consequently, the sea surface deformation in Fig. 2a fits the tsunami records [DART buoys (21418, 21401, 21413, 21419), GPS buoys (GPS801, GPS802, GPS803, GPS804, GPS 806, GPS807), pressure gauges (TM1, TM2) and cables (KPG1, KPG2, HPG)] as it was inverted from them (as well as from seismological and geodetic data)^2^.

### Global Positioning System (GPS) – Total Electron Content (TEC)

Each GPS satellite transmits information on two carrier frequencies. When these radio signals propagate through the ionosphere the carrier experiences a phase advance while the codes experience the propagation delay. The carrier-advance and code-delay are assumed to be proportional to total electron content (TEC) along the line of sight (LOS) from a satellite to receiver. The TEC along the LOS is widely known as the slant TEC (sTEC). We estimate the sTEC (Eq. 2) by analyzing the carrier-phase observed by PRNs 05 and 26 from Japan GEONET stations (Fig. 1) on 11 March 2011 during 5 to 7 UT.

2 $$sTEC=\frac{1}{40.3}\times \left(\frac{{f}_{1}^{2}\times {f}_{2}^{2}}{{f}_{1}^{2}-{f}_{2}^{2}}\right)({L}_{1}\times {\lambda }_{1}-{L}_{2}\times {\lambda }_{2})$$

where *f*_1_ is the carrier frequency of 1575.42 MHz*, λ*_1_ and *L*_1_ are the corresponding wavelength and carrier phase respectively. While *f*_2_ is the carrier frequency of 1227.60 MHz, *λ*_2_ and *L*_2_ are the corresponding wavelength and carrier phase respectively. Since GPS time is not in accordance with the UTC time, we adjust the estimated sTEC time for the leap second of 15 s. In order to reduce the uncertainty while identifying the perturbation onset, we avoid any filtering of the estimated sTEC. The onset of CIP is the time when the sTEC starts to increase or decrease suddenly. The onset can be found with very high precision from high-rate GPS data, which is the case here. The onset time presented in the study has been determined manually^8,16^. The LOS geometry, i.e. elevation and azimuth of a moving satellite at each epoch are calculated based on the navigation information as transmitted by each satellite and the station coordinates.

### 3D model for the evolution of seismo-acoustic rays in space and time

The seismo-acoustic rays could be described as wave perturbations of acoustic frequencies in the overlying atmosphere generated mainly by the crustal deformation surrounding an epicentral region. The acoustic wave propagation highly depends on the ambient temperature and density through their velocity (Eq. 3) which causes the refraction of seismo-acoustic rays in the neutral atmosphere as they propagate upward.

3 $${\rm{V}}=\sqrt{\frac{\gamma RT}{M}}$$

here, ${\rm{\gamma }}$ is the specific heat capacity, R is the universal gas constant, T is the temperature and M is the molecular mass density. Using this, we estimate the altitudinal variation of acoustic wave velocity over the Tohoku-Oki epicentre at the event onset time. The neutral atmospheric temperature and density are obtained from the NRLMSISE-00 model^32^. The varying atmospheric temperature with altitudes and corresponding acoustic wave velocity are shown in figure Suppli_3c. Using the estimated velocity profile, we model the evolution of seismo-acoustic rays in 3D space and time^24^. This model is developed based on the wave refraction phenomenon in varying velocity medium^40–42^. In the present case, we model the rays every 1 km of atmospheric altitude. More details on this could be found in Thomas *et al*.^24^.

### Seismic sources separation by ≥0.5°

The multiple seismic sources are demarcated as maximum water displacement fields (either uplift or subsidence) in a given patch separated by ≥0.5° in latitude or longitude. Further, we constraint the separation between the station-satellite azimuthal plane and the station-source azimuthal plane to be around ≤15° at the interaction time of satellite LOS and seismo-acoustic rays for most of the CIP presented here^24^. This azimuthal relaxation can account for the temporal uncertainty up to ~51 s while modelling the interaction time. From our analysis, for the obtained station-source distance and for the azimuthal relaxation up to ~15°, the satellite LOS could diverge up to ~50 km from the zenith of a given source (supplementary Table 1). Based on the adopted constraint, this is the maximum lateral uncertainty that may emerge in locating a source. Therefore, the sources are demarcated laterally by 0.5° (~55 km) or more. We prefer to apply this constraint while demarcating the distinct seismic sources in a given time window of 10 s, so the interactions between the seismo-acoustic rays from nearby sources can be avoided. It is worth to note that this constraint allowed us to model the interaction of PRN 05 and 26 LOS, from GPS stations in Fig. 1, with seismo-acoustic rays propagating from distinct seismic sources. That is, none of these LOSs interacted with the rays from more than one source in 480 s of the Tohoku-Oki event.

## Supplementary information

Supplementary movie 1.

Supplementary Information.

## Data Availability

The 1-Hz GPS data from GEONET is obtained from the Geospatial Information Authority of Japan.
