# Supplementary material for: The Ionospheric view of the 2011 Tohoku-Oki earthquake seismic source: the first 60 seconds of the rupture
Source: Sci Rep. 2020 Mar 23;10:5232. doi: 10.1038/s41598-020-61749-x (PMC7090075; doi:10.1038/s41598-020-61749-x)
Supplement: Supplementary file 2 — Supplementary Information. [file 41598_2020_61749_MOESM2_ESM.doc]

**Supplementary Information**

**The Ionospheric view of the 2011 Tohoku-Oki earthquake seismic source: the first 60 seconds of the rupture**

Mala S. Bagiya^1^, Dhanya Thomas^1^, Elvira Astafyeva^2^, Quentin Bletery^3^, Philippe Lognonné^2^, D. S. Ramesh^1^

^1^ Indian Institute of Geomagnetism (DST), Navi Mumbai, India

^2^ Institut de Physique du Globe de Paris, Université de Paris, CNRS UMR 7154, Paris

Cedex, France

^3^Université Côte d’Azur, IRD,CNRS, Observatoire de la Côte d’Azur, Géoazur, Sophia-Antipolis, Valbonne, France

*Corresponding Author: [bagiyamala@gmail.com](mailto:bagiyamala@gmail.com)

**Supplementary**

**Figures Suppli_1, Suppli_2, Suppli_3**

**Table 1**

**Movie 1**

**
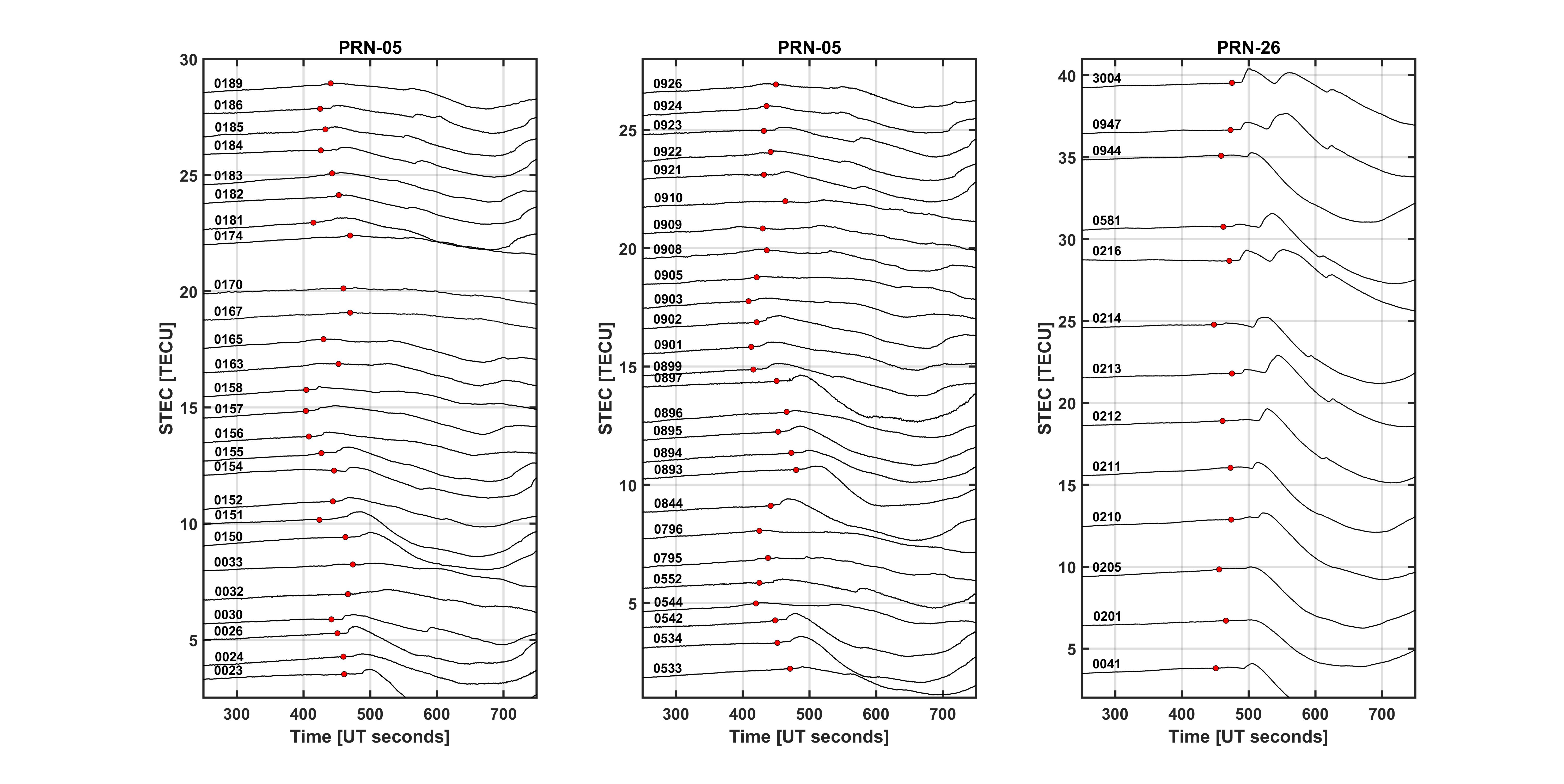
**

**Figure Suppli_1:** Enlarged CIP time series of figure 1b between 250 s from the earthquake onset to 750 s.

**
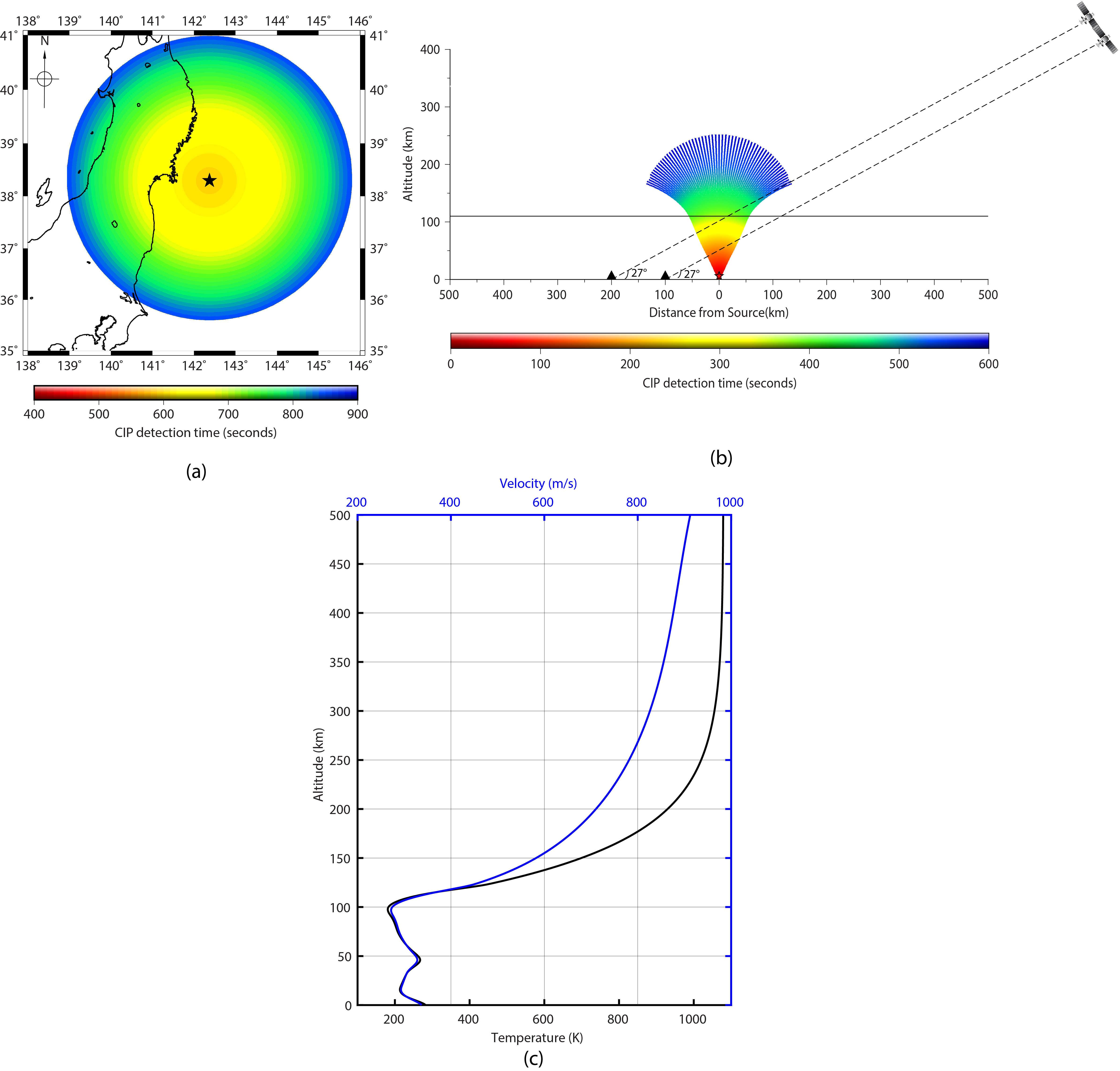
**

**­­Figure Suppli_2:** (a) Modelled arrival time of seismo-acoustic rays at ionospheric peak electron density altitude of 250 km. Epicentral projection at this altitude is also shown. (b) Vertical evolution of seismo-acoustic rays in a 2D plane. Triangles show two hypothetical GNSS stations. Realistic satellite LOS at an elevation of ~27^o^ from these stations shown in dotted line. (c) Neutral atmospheric temperature profile derived from the NRLMSISE-00 model^32^ along with the acoustic wave velocity variations estimated based on this temperature profile.

**
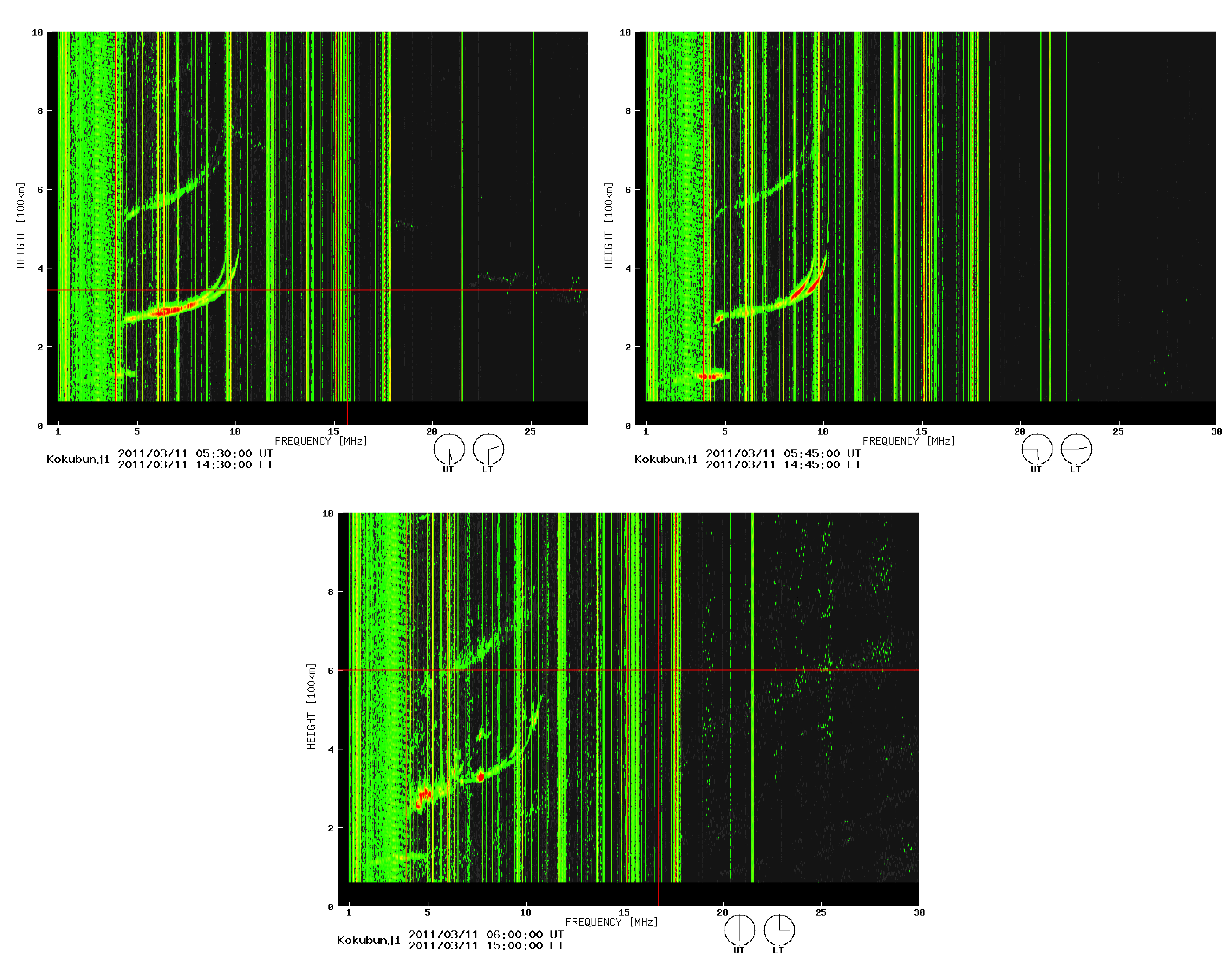
**

**Figure Suppli_3:** Ionograms recorded at Kokobunji ionosonde station on 11 March 2011. These ionograms correspond to the earthquake period, 15 min before the earthquake and 15 min after.

**Table 1**

Detailed output of our 3D model analysis, demonstrating the estimated detection altitude and the identified generative seismic sources of CIP shown in figure 1. The CIP detection time, satellite elevation and azimuth are estimated based on the GPS observations as described in the methodology section. The table has four segments. The first segment demonstrates the demarcated SG seismic sources and the corresponding CIP that detected by PRN 05. The second segment shows the identified SI seismic sources and the corresponding CIP that detected by PRN 05. The third and fourth segments are respectively the same as that of first and second but for PRN 26.

For the estimated station-source distance and separation between the station-source azimuthal plane and station-satellite LOS azimuthal plane, we further compute the divergence of LOS from the zenith of respective source. This is the maximum lateral uncertainty that may emerge in locating a seismic source (column 10).

| Station ID | CIP observed detection time (s) | Identified seismic source | Modelled time at the interaction between acoustic rays and satellite LOS (s) | Estimated detection altitude (km) | Azimuth (Station-Satellite LOS) | Azimuth (Station-Source) | Satellite Elevation | Station Distance from the identified seismic source (km) | Uncertainty in source location (km) |
| --- | --- | --- | --- | --- | --- | --- | --- | --- | --- |
| SG seismic sources (PRN 05) | | | | | | | | | |
| 181 | 415 | SG3 | 445 | 137 | 132.66 | 135.01 | 24.16 | 298.65 | 12.25 |
| 184 | 426 | SG1 | 426 | 131 | 132.11 | 135.97 | 23.81 | 282.24 | 19.01 |
| 186 | 425 | SG1 | 425 | 130 | 132.02 | 131.73 | 24.10 | 253.17 | 1.29 |
| 544 | 420 | SG5 | 420 | 126 | 132.33 | 132.28 | 24.98 | 253.09 | 0.20 |
| 552 | 425 | SG1 | 425 | 129 | 131.87 | 128.46 | 24.13 | 250.97 | 14.94 |
| 908 | 437 | SG2 | 437 | 132 | 132.59 | 132.94 | 24.75 | 234.76 | 1.45 |
| 921 | 432 | SG1 | 432 | 133 | 132.17 | 137.10 | 23.69 | 290.89 | 25.11 |
| 922 | 442 | SG1 | 442 | 131 | 132.51 | 140.00 | 23.98 | 256.54 | 33.70 |
| 924 | 436 | SG2 | 436 | 138 | 132.48 | 135.27 | 24.29 | 281.52 | 13.74 |
| 926 | 450 | SG5 | 450 | 140 | 132.37 | 132.78 | 24.55 | 275.84 | 2.01 |
| 182 | 453 | SG1 | 453 | 138 | 132.46 | 139.26 | 23.75 | 271.91 | 32.44 |
| 189 | 441 | SG5 | 441 | 134 | 132.14 | 130.93 | 24.45 | 315.73 | 6.67 |
| 796 | 425 | SG5 | 425 | 123 | 132.45 | 130.96 | 25.25 | 246.07 | 6.42 |
| 170 | 460 | SG7 | 460 | 122 | 133.52 | 144.33 | 25.43 | 211.54 | 40.38 |
| 910 | 464 | SG5 | 464 | 129 | 133.01 | 138.62 | 25.11 | 237.45 | 23.28 |
| 905 | 421 | SG10 | 421 | 117 | 133.61 | 133.80 | 25.06 | 233.68 | 0.77 |
| 154 | 446 | SG1 | 455 | 140 | 132.25 | 139.19 | 23.36 | 316.34 | 38.49 |
| 183 | 443 | SG3 | 443 | 136 | 132.93 | 135.96 | 24.18 | 277.44 | 14.70 |
| 163 | 453 | SG6 | 453 | 124 | 133.16 | 141.91 | 24.63 | 231.62 | 35.63 |
| 923 | 432 | SG1 | 432 | 135 | 132.01 | 133.00 | 23.85 | 273.45 | 4.69 |
| 185 | 433 | SG1 | 433 | 129 | 132.37 | 136.93 | 24.17 | 242.22 | 19.30 |
| 165 | 430 | SG2 | 430 | 129 | 132.77 | 137.67 | 24.77 | 238.60 | 20.44 |
| 30 | 442 | SG1 | 442 | 139 | 131.75 | 127.91 | 23.74 | 278.77 | 18.70 |
| 157 | 405 | SG3 | 423 | 112 | 133.11 | 141.20 | 24.56 | 267.49 | 38.00 |
| 909 | 430 | SG3 | 430 | 116 | 132.98 | 139.22 | 25.16 | 256.88 | 28.05 |
| 795 | 438 | SG10 | 438 | 122 | 133.40 | 128.34 | 24.91 | 238.22 | 21.06 |
| 167 | 470 | SG11 | 470 | 123 | 133.84 | 145.30 | 25.31 | 223.03 | 45.19 |
| 155 | 427 | SG18 | 427 | 112 | 132.81 | 130.46 | 23.93 | 240.66 | 9.90 |
| 897 | 451 | SG18 | 476 | 127 | 132.94 | 136.92 | 23.14 | 303.97 | 21.13 |
| 26 | 451 | SG18 | 451 | 119 | 132.73 | 131.27 | 23.39 | 278.67 | 7.09 |
| 151 | 424 | SG18 | 460 | 112 | 133.14 | 140.22 | 23.59 | 278.41 | 34.57 |
| 534 | 452 | SG18 | 482 | 124 | 133.18 | 140.28 | 23.20 | 298.32 | 37.14 |
| 844 | 442 | SG18 | 442 | 113 | 133.28 | 139.53 | 23.73 | 251.17 | 27.48 |
| SI seismic sources (PRN 05) | | | | | | | | | |
| 33 | 474 | SI3 | 474 | 136 | 131.76 | 131.53 | 24.77 | 258.04 | 1.05 |
| 32 | 468 | SI3 | 477 | 133 | 131.29 | 127.82 | 24.38 | 301.72 | 18.31 |
| 533 | 471 | SI4 | 471 | 127 | 134.36 | 139.99 | 23.48 | 273.87 | 26.97 |
| 896 | 466 | SI4 | 466 | 127 | 134.15 | 135.18 | 23.67 | 257.32 | 4.61 |
| 893 | 480 | SI4 | 508 | 148 | 133.61 | 133.51 | 22.64 | 350.81 | 0.58 |
| 24 | 460 | SI4 | 460 | 125 | 134.02 | 135.21 | 23.50 | 278.56 | 5.80 |
| 152 | 444 | SI4 | 444 | 118 | 133.94 | 131.36 | 23.96 | 243.70 | 11.00 |
| 894 | 473 | SI4 | 473 | 130 | 134.03 | 136.27 | 23.21 | 298.63 | 11.63 |
| 895 | 453 | SI4 | 460 | 121 | 133.70 | 130.74 | 23.49 | 284.92 | 14.73 |
| 150 | 463 | SI4 | 480 | 133 | 133.77 | 133.59 | 23.16 | 310.40 | 0.94 |
| 542 | 449 | SI2 | 458 | 128 | 132.98 | 132.02 | 23.41 | 295.96 | 4.97 |
| 899 | 416 | SI2 | 416 | 112 | 133.35 | 134.89 | 24.28 | 230.71 | 6.22 |
| 901 | 413 | SI2 | 432 | 114 | 133.53 | 137.18 | 24.50 | 211.77 | 13.49 |
| 902 | 421 | SI2 | 421 | 112 | 133.12 | 129.78 | 24.25 | 231.07 | 13.51 |
| 158 | 404 | SI1 | 433 | 112 | 133.84 | 142.19 | 24.87 | 224.35 | 32.91 |
| 156 | 408 | SI1 | 437 | 114 | 133.70 | 140.66 | 24.64 | 244.57 | 29.82 |
| 903 | 409 | SI1 | 428 | 115 | 133.55 | 135.84 | 24.80 | 225.78 | 9.04 |
| 23 | 461 | SI2 | 510 | 153 | 132.90 | 135.38 | 22.66 | 363.57 | 15.76 |
| 174 | 470 | SI3 | 469 | 127 | 132.23 | 138.64 | 25.12 | 227.63 | 25.56 |
| SG seismic sources (PRN 26) | | | | | | | | | |
| 210 | 474 | SG4 | 483 | 141 | 56.60 | 51.99 | 40.22 | 214.85 | 17.31 |
| 944 | 459 | SG4 | 459 | 134 | 56.58 | 45.96 | 40.70 | 180.15 | 33.76 |
| 205 | 456 | SG1 | 456 | 143 | 56.71 | 49.79 | 40.77 | 186.91 | 22.66 |
| 41 | 451 | SG2 | 462 | 144 | 56.44 | 55.56 | 40.83 | 201.37 | 3.07 |
| 201 | 466 | SG1 | 466 | 152 | 57.03 | 54.96 | 40.94 | 164.44 | 5.94 |
| 211 | 473 | SG4 | 473 | 137 | 56.58 | 46.45 | 40.44 | 197.23 | 35.22 |
| 212 | 461 | SG2 | 518 | 175 | 56.25 | 56.11 | 40.24 | 251.61 | 0.61 |
| SI seismic sources (PRN 26) | | | | | | | | | |
| 581 | 462 | SI3 | 510 | 137 | 56.14 | 73.45 | 40.22 | 184.97 | 57.63 |
| 214 | 448 | SI3 | 487 | 129 | 56.11 | 73.47 | 40.53 | 161.30 | 50.40 |
| 213 | 475 | SI3 | 532 | 149 | 56.13 | 72.05 | 39.93 | 205.46 | 58.60 |
| 947 | 473 | SI3 | 511 | 142 | 55.94 | 66.20 | 39.90 | 204.63 | 37.02 |
| 216 | 471 | SI3 | 500 | 138 | 55.80 | 61.56 | 39.89 | 203.86 | 20.57 |
| 3004 | 475 | SI3 | 498 | 135 | 55.67 | 56.41 | 39.82 | 207.97 | 2.69 |

**Movie 1**

Cumulative ocean water displacement estimated every 5 s from the onset to 60 s of the Tohoku-Oki event. CIP evolution at the respective detection altitudes (that estimated in this study) every 5 s from the onset of first CIP i.e. ~404 s of the event. CIP evolution is presented till 480 s of the event.
